# Evidence for Unknown *Sarcocystis*-Like Infection in Stranded Striped Dolphins (*Stenella coeruleoalba*) from the Ligurian Sea, Italy

**DOI:** 10.3390/ani11051201

**Published:** 2021-04-22

**Authors:** Federica Giorda, Umberto Romani-Cremaschi, Antoinette E. Marsh, Carla Grattarola, Barbara Iulini, Alessandra Pautasso, Katia Varello, Enrica Berio, Paola Gazzuola, Letizia Marsili, Cristina E. Di Francesco, Maria Goria, Federica Verna, Tania Audino, Simone Peletto, Maria Caramelli, Mercedes Fernández-Escobar, Eva Sierra, Antonio Fernández, Rafael Calero-Bernal, Cristina Casalone

**Affiliations:** 1 Istituto Zooprofilattico Sperimentale del Piemonte, Liguria e Valle d’Aosta, 10154 Torino, Italy; federica.giorda@izsto.it (F.G.); carla.grattarola@izsto.it (C.G.); barbara.iulini@izsto.it (B.I.); katia.varello@izsto.it (K.V.); enrica.berio@izsto.it (E.B.); paola.gazzuola@izsto.it (P.G.); maria.goria@izsto.it (M.G.); tania.audino@izsto.it (T.A.); simone.peletto@izsto.it (S.P.); maria.caramelli@izsto.it (M.C.); 2 Institute for Animal Health and Food Safety (IUSA), Veterinary School, University of Las Palmas de Gran Canaria, Las Palmas de Gran Canaria, 35416 Canary Islands, Spain; eva.sierra@ulpgc.es (E.S.); antonio.fernandez@ulpgc.es (A.F.); 3 Veterinary Department, Mundomar, Calle Sierra Helada s/n, 03503 Benidorm, Spain; umberto.romanicremaschi@gmail.com; 4 Department of Veterinary Preventive Medicine, College of Veterinary Medicine, The Ohio State University, 1920 Coffey Road, Columbus, OH 43210, USA; marsh.2061@osu.edu; 5 Department of Prevention, Local Veterinary Services (ASL1 Imperiese), Via Aurelia Ponente 97, Bussana di Sanremo, 18038 Imperia, Italy; a.pautasso@asl1.liguria.it; 6 Dipartimento di Scienze Fisiche, della Terra e dell’Ambiente, University of Siena, Via Mattioli 4, 53100 Siena, Italy; letizia.marsili@unisi.it; 7 Faculty of Veterinary Medicine, University of Teramo, Strada Provinciale 18 Località Piano d’Accio, 64100 Teramo, Italy; cedifrancesco@unite.it; 8 Department of Prevention, Local Veterinary Services, Via Conte Verde 125, 35040 Asti, Italy; fverna@asl.at.it; 9 SALUVET, Department of Animal Health, Faculty of Veterinary, Complutense University of Madrid, 28040 Madrid, Spain; merfer02@ucm.es (M.F.-E.); r.calero@ucm.es (R.C.-B.)

**Keywords:** striped dolphin, tissue cysts, neuropathology, *Toxoplasma gondii*, *Sarcocystis*-like, genotype

## Abstract

**Simple Summary:**

Two stranded striped dolphins presented meningoenchepalitic lesions associated with the presence of unknown protozoan tissue cysts. The present study aimed at fully characterizing these previously undescribed parasites. Light microscopy re-examination of affected CNS areas showed high numbers of tissue cysts with morphological features resembling those of *Sarcocystis* species. Tissue cyst bradyzoites positively stained when labeled with polyclonal antisera but cross-reactivity could not be precluded. *Sarcocystis* sp. sequences with high homology to species infecting livestock were amplified by means of PCR from myocardial and muscle tissues. This is the first report of *Sarcocystis*-like tissue cysts in the cerebral tissue of stranded cetaceans with muscular sarcocystosis in Mediterranean dolphins. The obtained results may suggest a land-to-sea cycling of Apicomplexan parasites in this region and the need for further investigations in order to foster marine mammal conservation.

**Abstract:**

Two striped dolphins (SD1, SD2), stranded along the Ligurian coast of Italy, were diagnosed with a nonsuppurative meningoencephalitis associated with previously undescribed protozoan tissue cysts. As tissue cysts were morphologically different from those of *Toxoplasma gondii*, additional histopathological, immunohistochemical, ultrastructural, and biomolecular investigations were performed, aiming to fully characterize the organism. Histopathology revealed the presence of large *Sarcocystis*-like tissue cysts, associated with limited inflammatory lesions in all CNS areas studied. IHC was inconclusive, as positive staining with polyclonal antisera did not preclude cross-reaction with other Sarcocystidae coccidia. Applied to each animal, 11 different PCR protocols precluded a neural infection by *Sarcocystis neurona, Sarcocystis falcatula, Hammondia hammondi*, and *Neospora caninum*. *T. gondii* coinfection was confirmed only in dolphin SD2. *Sarcocystis* sp. sequences, showing the highest homology to species infecting the Bovidae family, were amplified from SD1 myocardium and SD2 skeletal muscle. The present study represents the first report of *Sarcocystis*-like tissue cysts in the brain of stranded cetaceans along with the first description of *Sarcocystis* sp. infection in muscle tissue of dolphins from the Mediterranean basin.

## 1. Introduction

Tissue cyst-forming coccidia from the genera *Toxoplasma*, *Sarcocystis*, and *Neospora* (Apicomplexa) are capable of infecting several species of marine mammals and are responsible for either chronic diseases or acute mortality [1]. While *Neospora caninum* does not seem to pose a major threat to marine wildlife, *Toxoplasma gondii* and *Sarcocystis neurona* are the two coccidian parasites most widely reported in North American marine mammals [1], especially in coastal species that are more likely to be overexposed to immunosuppressant chemical pollutants and to high concentrations of land-derived oocysts [2,3,4]. The most probable exposure route in these animals is through ingestion of environmentally resistant oocysts or sporocysts shed on land by the definitive hosts and passed into the sea through freshwater runoffs or the release of contaminated ship waters. Eventually, protozoal infectious stages may then accumulate in marine invertebrates, bivalve mollusks, or fish on which intermediate hosts prey [5]. In the Mediterranean basin, *T. gondii* is a frequent finding in stranded odontocetes, and it is often associated with protozoal meningoencephalitis [6,7].

On the other hand, neither muscular nor neural sarcocystosis has ever been officially reported in this geographical area, to the authors’ best knowledge. However, a fatal case of hepatic sarcocystosis [8], caused by an unknown species, is the only account of a *Sarcocystis* sp. infection in a Mediterranean cetacean.

In this study, we provide evidence of an infection sustained by a *Sarcocystis*-like organism in two striped dolphins (*Stenella coeruleoalba*) stranded along the Ligurian coast of Italy, in the marine protected area of the Pelagos Sanctuary, in 2011 and 2017.

## 2. Materials and Methods

### 2.1. Naturally Infected Dolphins

**Figure 1:**
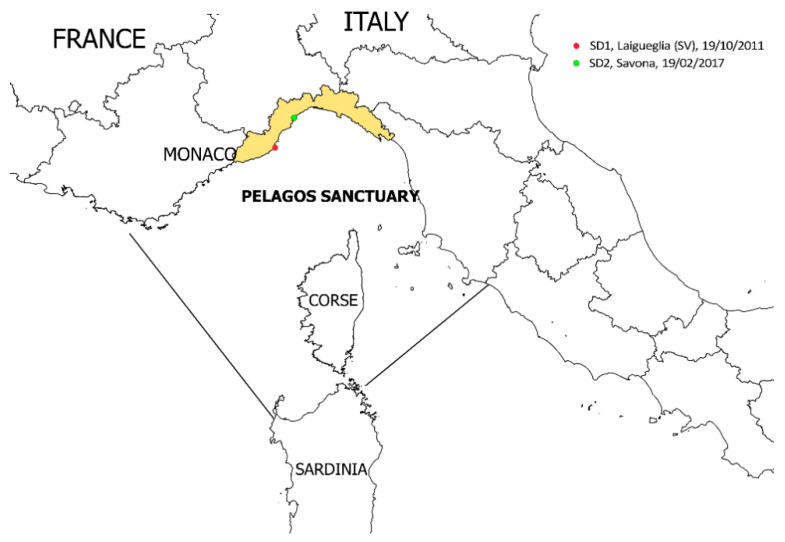
Map of the study area (Ligurian coastline), displaying the stranding locations (red and green dots) of the two striped dolphins infected by Sarcocystidae organisms. The map was created by A.P. with QGIS (QGIS Development Team (2018). QGIS Geographic Information System. Open Source Geospatial Foundation Project. http://qgis.osgeo.org, accessed on 17 August 20).

The cases, SD1 and SD2, included in the present study were diagnosed during routine pathological and cause-of-death assessment in stranded cetaceans at the Istituto Zooprofilattico Sperimentale del Piemonte, Liguria e Valle d’Aosta. The two striped dolphins (*Stenella coeruleoalba*), stranded along the Ligurian Sea coast in 2011 (SD1) and 2017 (SD2) (Figure 1), were submitted for a complete postmortem examination, according to standard protocols [9]. Dolphin SD1 was a 206 cm (total length, TL) adult male, in a poor nutritional status and in a postmortem condition code 3 (moderately decomposed). Dolphin SD2 was a 177 cm (TL) juvenile male, in a moderate nutritional status and in postmortem condition code 2 (fresh). Neither animals displayed any evidence of interaction with fishing activities, and the stomach chambers were devoid of intake. Initially, postmortem findings from SD1 and SD2 were previously published [10,11] and, for the present study, these two cases were re-examined with the focus on neurotrophic causes of inflammation, CNS, and tissue protozoal cysts, parasite identification, and distribution.

During necropsy, the tissue samples of all the major organs and lesions were collected and split into aliquots for subsequent analyses: one was kept frozen at −20 °C for microbiological and toxicological investigations, one was kept frozen at −80 °C for biomolecular analyses, and the other was preserved in 10% buffered formalin for histological and immunohistochemical (IHC) investigations. Blood serum, aqueous humor, and cerebrospinal fluid (CSF) were collected, when available, and kept frozen at −20 °C for serological investigations.

### 2.2. Histology and Immunohistochemistry

Representative tissues from SD1 (brain, lung, heart, liver, spleen, kidney, prescapular lymph node, urinary bladder, and reproductive system) and from SD2 (brain, tonsils, lung, prescapular and tracheobronchial lymph nodes, heart, liver, spleen, pancreas, intestine, skeletal muscle, skin, kidney, urinary bladder, adrenal gland, tongue lesion, and reproductive system) were collected and fixed in 10% neutral buffered formalin, embedded in paraffin, sectioned at 4 ± 2 μm, stained with hematoxylin and eosin (H&E), and examined through a light microscope.

Nine different areas from the brain were sampled and examined, including basal nuclei, thalamus, mesencephalon, pons, obex, and frontal, parietal, occipital, and cerebellar cortex. Immunohistochemistry (IHC) for *Morbillivirus* was performed on tissue sections from SD1 (brain) and from SD2 (brain, tonsils, lung, prescapular and pulmonary lymph nodes, spleen, kidney, urinary bladder, liver, skin, and muscle), using a monoclonal anti-*Canine distemper virus* (CDV) antibody (VMRD, Pullman, WA, USA) [6]. *Toxoplasma gondii* IHC was carried out on the nine aforementioned brain tissues of each case, using a polyclonal serum of caprine origin (VMRD, Pullman, WA, USA) [6].

### 2.3. PCR and Sequence Analysis

Molecular detection of *Dolphin morbillivirus* (DMV) [12], *Herpesvirus* (HV) [13], *T. gondii* [14], and *Brucella* spp. [15] was routinely achieved on target tissues available from each case, consisting of brain, lung, tonsils, lymph nodes, liver, spleen, kidney, bladder, and blood for DMV, brain, lung, lymph nodes, spleen, and kidney for HV, brain, lymph nodes, liver, spleen, heart, and muscle for *T. gondii*, and brain, lung, tonsils, lymph nodes, liver, spleen, kidney, and blood for *Brucella* spp.

For DMV assays, amplicons were directly sequenced using PCR primers on a 3130XL Genetic Analyzer (Thermo Fisher Scientific Inc., Waltham, MA, USA). Sequences were aligned using the SeqMan software (Lasergene package. DNASTAR Inc., Madison, WI, USA) to obtain a consensus sequence and compared with available sequences retrieved from the National Center for Biotechnology Information (NCBI) database through the BLAST tool (http://blast.ncbi.nlm.nih.gov/Blast.cgi, accessed on 20 February 2021).

### 2.4. Serological, Toxicological, and Microbiological Analyses

Serological investigations to screen for the presence of specific antibodies against DMV and *T. gondii* were performed [6] on serum, CSF, and aqueous humor, when these samples were available from SD1 and SD2. These same samples were also tested by rapid serum agglutination (Rose Bengal plate test, RBT) using RBT antigen produced from *B. abortus* strain S99 [6,16] to detect anti-smooth *Brucella* spp. antibodies.

Toxicological investigations were carried out only in SD2. The toxicological analysis did not include tissues from SD1 as they were collected before implementing a sampling protocol that included contaminant analyses. Polychlorinated biphenyls (PCBs), hexachlorobenzene (HCB), and dichlorodiphenyltrichloroethanes (DDTs) were measured in blubber. Measurements were made according to Environmental Protection Agency method 8081/8082, with modifications [17], and toxicological stress was evaluated using a theoretical model [18].

Tissue samples including brain, lung, lymph nodes, liver, spleen, and kidney (SD1 and SD2) were processed for standard aerobic, anaerobic, and microaerobic (5% CO_2_) bacterial culture and identification, using biochemical and/or molecular analyses. Following international recommendations [19], samples from target tissues underwent specific bacteriological procedures to screen for *Salmonella* spp., *Listeria* spp., and *Brucella* spp.

### 2.5. Light Microscopy Re-Examination for Parasite Characterization: Histology and Immunohistochemistry

CNS and heart sections from both SD1 and SD2 and skeletal muscle tissue only from SD2 were hematoxylin and eosin (H&E)-stained for light microscopy re-examination. For each organ and the aforementioned CNS areas studied, nine additional 5 µm thick sections were cut in series from stored paraffin blocks. Sections n° 1, 4, and 7 were stained with H&E for light microscopy examination. For IHC, only sections adjacent to the H&E slides presenting tissue cysts which were clearly distinct from *T. gondii* tissue cysts were used (well-defined cyst wall and >8 µm long bradyzoites). The IHC protocols included *S. neurona* polyclonal antibodies (PoAb Rabbit 1 R81, [20]), *S. falcatula* polyclonal antibodies (PoAb Rabbit 2 R-anti SF, [20]), and *S. neurona* monoclonal antibody (MAb 2G5, [21]). The IHC analyses were performed as previously described [21,22]. *Sarcocystis neurona*-infected and noninfected murine brains from interferon gamma (IFN-γ) knockout B6.129S7-Ifngtm1Ts (*Mus musculus*) (Jackson Laboratories, Bar Harbor, ME, USA) mice [23] were used, respectively, as positive and negative controls for the polyclonal antibodies, whereas *S. neurona*-infected opossum (*Didelphis virginiana*) intestine tissues and the brain of a bottlenose dolphin (*Tursiops truncatus*) foetus born under human care were used, respectively, as positive and negative control for the monoclonal antibody.

### 2.6. Electron Microscopy Examination

Transmission electron microscopy (TEM) was performed on CNS samples of both specimens at the Spanish National Center for Electron Microscopy (Complutense University of Madrid). For each animal, two cysts were excised from the paraffin block and samples were prepared for TEM as previously described [24]. Ultra-thin sections were cut on a Leica UC6 ultramicrotome (Leica Microsystems GmbH, Wetzlar, Germany), mounted onto TEM grids, and stained with 6% saturated uranyl acetate and 3% lead citrate. Sections were examined with a JEOL JEM 1400 Plus (JEOL USA Inc., Peabody, MA, USA) transmission electron microscope operated at 80 kV.

### 2.7. Molecular Analyses: Parasite Detection, Identification, and Characterization

Genomic DNA was extracted from CNS, myocardium, and skeletal muscle tissue samples from SD1 and SD2 and screened for the presence of tissue-cyst forming coccidia (*Sarcocystis* spp., *T. gondii* and *N. caninum*) DNA using 11 different PCR protocols, detailed in Supplementary material Table S1. DNA extraction from frozen samples was achieved using a “four-step” method, namely, ReliaPrep™ gDNA Tissue Miniprep System (Promega Italia S.r.l. Milan, Italy), whereas DNA extraction from paraffin-embedded samples, previously purified using the QIAamp DNA FFPE Tissue Kit, was achieved using QIAamp^®^ DNA Mini Kit (QIAGEN, Hilden, Germany).

For each positive PCR result, amplicons of the expected size were sequenced using the BigDye^®^ Terminator kit v 3.1 (Applied Biosystems, Foster City, CA, USA) and analyzed on an ABI 3130 Genetic Analyzer (Applied Biosystems). The obtained sequences were curated manually if necessary and analyzed using BioEdit software, version 7.0.5.3 [25]. Generated DNA consensus sequences were aligned to appropriate reference sequences using MEGA X software (http://www.megasoftware.net/ accessed on 20 April 2021) [26], and compared with available sequences retrieved from the NCBI database through the BLAST tool (http://blast.ncbi.nlm.nih.gov/Blast.cgi, accessed on 20 February 2021).

*Toxoplasma gondii* strain genotyping analyses were carried out at the Complutense University of Madrid. DNA extracts from SD2 heart, muscle, and brain tissues were subjected to the widely used Mn-PCR restriction fragment length polymorphism (RFLP) method, with the markers *SAG1, SAG2 (5′–3’ SAG2,* and alt. *SAG2), SAG3, BTUB, GRA6, c22-8, c29-2, L358, PK1, Apico*, and *CS3* [27,28]. ToxoDB RFLP genotype was identified according to http://toxodb.org/toxo/ accessed on 20 April 2021.

## 3. Results

### 3.1. Naturally Infected Dolphins

Results of *postmortem*, routine investigations, along with anamnestic data, are summarized in Table 1.

### 3.2. Histology and Immunohistochemistry

Significant histopathological lesions detected for SD1 and SD2 are detailed in Table 1. Both SD1 and SD2 were diagnosed with severe and diffuse NS meningoencephalitis in association with the detection of protozoan tissue cysts (a single cyst for SD1 and two cysts for SD2). The cysts were morphologically distinct from *T. gondii*. In addition, SD2 was also diagnosed with *T. gondii*, whereas SD1 samples demonstrated no detectable antibodies for *T. gondii* nor detectable *T. gondii* DNA by PCR. No cysts were observed in the other target tissues investigated: muscle of SD2 and heart of both SD1 and SD2. *Morbillivirus*-specific antigens were detected in brain, urinary bladder, and muscle of SD2 by IHC, while SD1 did not show any specific staining.

All brain cysts observed in both animals stained positive with the polyclonal Ab raised for *T. gondii*. Since these data were not supported by molecular and serological investigations performed in SD1, an antigenic cross-reactivity among genetically related protozoa was hypothesized.

### 3.3. PCR and Sequence Analysis

No biomolecular evidence of DMV, HV, *T. gondii* or *Brucella* spp. was found in SD1.

A systemic DMV infection was demonstrated in SD2, through PCR, in brain, lung, laryngeal tonsils, tracheobronchial lymph node, spleen, kidney, and bladder, and subsequently confirmed through amplicon sequencing and BLAST analysis.

However, in SD2, molecular data supported a coinfection with both *T. gondii*, in brain, liver, muscle, spleen, and tracheobronchial and pulmonary lymph nodes, and *Brucella* sp., by PCR detection, in brain, liver, lung, spleen, and tracheobronchial, pulmonary, and prescapular lymph nodes. The HV analysis was negative for SD2.

### 3.4. Serological, Toxicological, and Microbiological Analyses

Anti-*Morbillivirus* antibodies (1:16) were detected in serum of SD1, while anti-*Morbillivirus* (1:8 serum) and anti-*T. gondii* antibodies (>1:640 serum; 1:160 CSF; 1:80 aqueous humor) were detected in SD2. No evidence of anti-*Brucella* spp. antibodies was demonstrated in sera, cerebrospinal fluid, or aqueous humor samples from either of the dolphins.

For SD2, the levels of PCBs, HCB, and DDTs, expressed in ng·g^−1^ on a lipid weight basis (PCBs: 136810.3; DDTs: 69866.92; HCB: 153.6; canonical variable value (CAN) = 0.688), confirmed the presence of immunotoxic levels of OC pollutants (CAN > 0.47).

*Photobacterium damselae* subsp. *damselae* was isolated by microaerobic bacterial culture from blowhole and lungs of SD1; no other significant bacteria, including *Salmonella* spp., *Listeria* spp., and *Brucella* spp., were isolated. *Brucella ceti* was isolated from CNS, spleen, and lung of SD2; no other significant bacteria, including *Listeria* spp. and *Salmonella* spp., were isolated.

### 3.5. Light Microscopy Re-Examination: Histology and Immunohistochemistry

**Figure 2:**
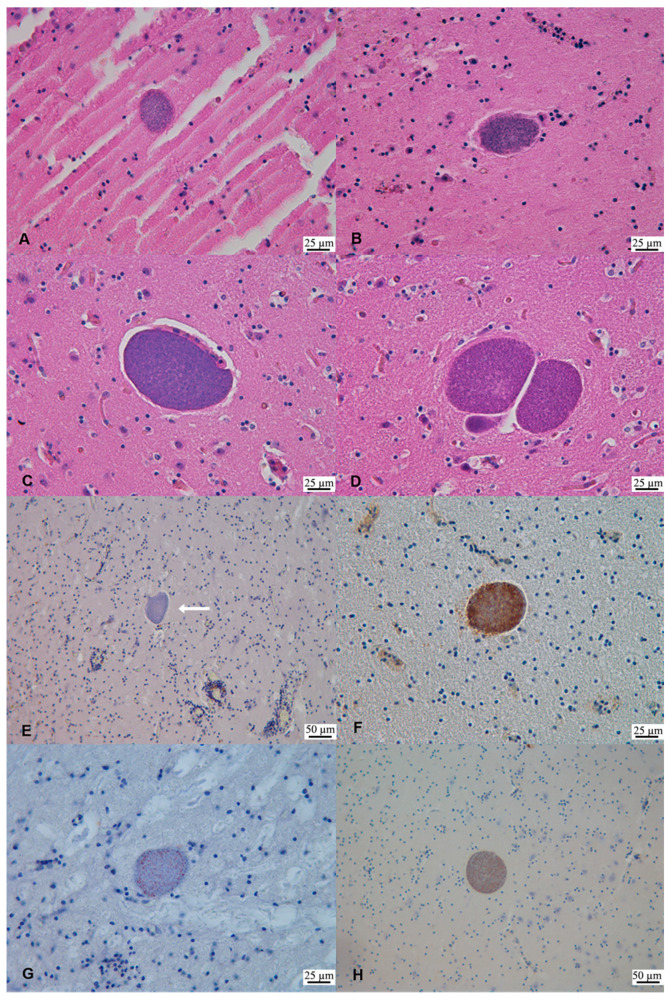
*Sarcocystis*-like tissue cysts in the brain of striped dolphins (*Stenella coeruleoalba*) SD1 and SD2 from Liguria, Italy. (**A**) Parietal cortex (SD1). Protozoan tissue cyst measuring 70 × 50 µm. H&E. (**B**) Occipital cortex (SD1). Protozoan tissue cyst measuring 44.6 × 58.1 µm. H&E. (**C**) Frontal cortex (SD2). Protozoan tissue cyst measuring 72.83 × 116.34 µm. H&E. (**D**) Basal ganglia (SD2). Protozoan tissue cysts measuring (left-right reading) 110 × 119.3 µm, 40 × 19.8 µm and 50 × 99.1 µm. H&E. (**E**) Mesencephalon (SD1). Negative immunostaining of a protozoan tissue cyst (arrow). Monoclonal Ab anti-*S. neurona*. (**F**) Cerebellum (SD1). Positive labeling of *Sarcocystis*-like tissue cyst. Polyclonal Ab anti-*S. falcatula.* (**G**) Mesencephalon (SD1). Positive labeling of a protozoan tissue cyst bradyzoites. Polyclonal Ab anti-*S. neurona.* (**H**) Parietal cortex (SD2). Positive immunostaining of a protozoan tissue cyst bradyzoites. Polyclonal Ab anti-*S. neurona.*

For both SD1 and SD2, several non-*Toxoplasma* protozoal tissue cysts were observed in all the CNS areas studied, with major involvement of the brain cortical areas. Overall, neural tissue cysts were round to oval in shape, from 27 to 119.3 µm in diameter, presented with a distinguishable, apparently smooth outer wall, and they were filled with mild basophilic bradyzoites (Figure 2). Within the range of the available magnifications, neither internal septations nor villous protrusions were visible by light microscopy. Moreover, it was not possible to detect the presence of free schizonts or merozoites in any of the brain sections examined. The histomorphological appearance was consistent with that of other apicomplexan coccidia, with most features resembling the genus *Sarcocystis.* No other cysts were observed in the heart of either case, while, in the skeletal muscle from SD2, only one tissue cyst morphologically resembling *T. gondii* was observed.

*Sarcocystis*-like tissue cysts in the brains of both SD1 and SD2 stained immunopositive with the anti-*S. neurona* polyclonal antiserum. Similar results were obtained with the anti-*S. falcatula* polyclonal antiserum whereas MAb 2G5 failed in labeling protozoal antigens. Overall, the application of the polyclonal antisera resulted in a negative staining of the cyst wall and in a sparse labeling of the enclosed bradyzoites (Figure 2).

### 3.6. Ultrastructural Description of Tissue Cysts

**Figure 3:**
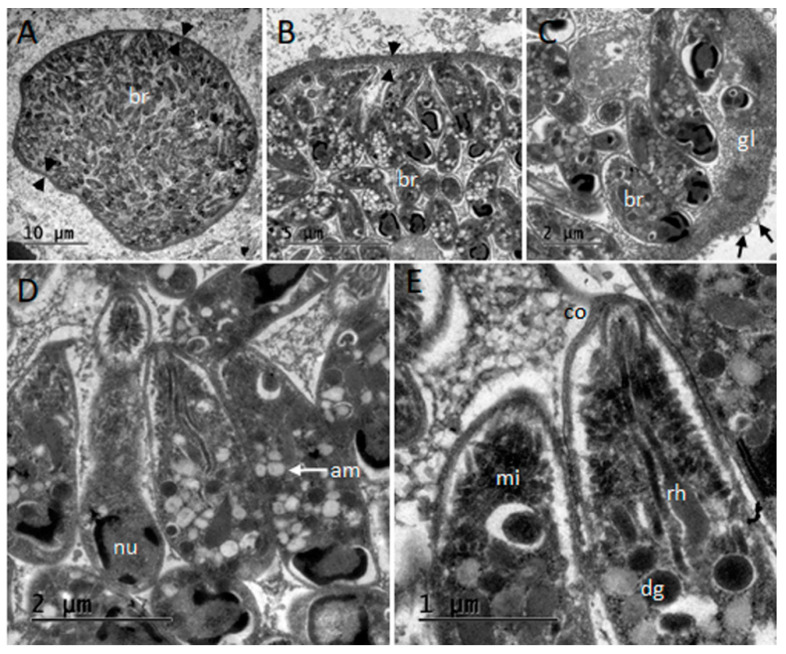
Transmission electron microscopy micrographs of the *Toxoplasma gondii* tissue cyst studied from central nervous system of striped dolphin (*Stenella coeruleoalba*) SD2 in Liguria, Italy. (**A**) Section of the thin-walled tissue cyst; note the cyst wall (arrowheads) and densely packaged bradyzoites (br). (**B**,**C**) Details of the simple and thin cyst wall (arrowheads) and granular layer (gl) presenting vesicles (arrows). (**D**,**E**) Ultrastructural details of bradyzoites, note: nucleus (nu), micronemes (mi), dense granules (dg), amylopectin granules (am), conoid (co), and rhoptries (rh).

After processing of samples, no tissue cysts could be observed from the SD1 specimen-derived blocks, but a mature thin-walled (400 nm) cyst resembling *T. gondii* (Figure 3) was examined in dolphin SD2. Typical morphology with simple wall structure presenting vesicles, absence of septae, and small bradyzoites of 5.3 × 1.4 µm in size (*n* = 10) were observed.

### 3.7. Molecular Detection and Parasite Identification

The findings on PCR screening for tissue-cyst forming coccidia DNA are summarized in Supplementary material Table S2. PCR n.1 (18S region) and n.9 protocols showed the presence of cyst-forming coccidia DNA in at least one tissue of each animal, in agreement with histological findings. Sequencing of PCR products confirmed the absence of *T. gondii* DNA in SD1 and the presence of *T. gondii* in SD2. PCR n.5 and n.6 protocols showed the presence of *Sarcocystis* sp. DNA in the tissues from both SD1 and SD2. The *Sarcocystis* sp. DNA detection was confirmed by sequencing of the amplicons produced from DNA extracted from SD1 myocardial tissue and SD2 skeletal muscle tissue. BLAST^®^ analysis retrieved different homology to *Sarcocystis* species infecting members of the family Bovidae, *S. hirsuta* (99.4%), and *S. buffalonis* (97.8%), respectively. These sequences were deposited in GenBank^®^ with the following accession numbers: MW151248 (*Sarcocystis* sp.) and MW151249 (*Sarcocystis* sp.).

PCR n.7 and n.8 protocols resulted in the detection of Sarcocystidae-unspecific products in heart and brain tissues from SD2 followed by PCR n.8, and DNA sequencing which was used to confirm the presence of *T. gondii* DNA in SD2 heart and brain tissues. This finding was further documented by PCR n.10 (specific for *T. gondii* amplification). Molecular methods confirmed the presence of *T.gondii* in addition to the detection of *Sarcocystis* DNA. The molecular results suggest a potentially greater distribution of *T. gondii* parasites or higher concentration of DNA from *T. gondii* as compared to the *Sarcocystis* sp.

**Figure 4:**
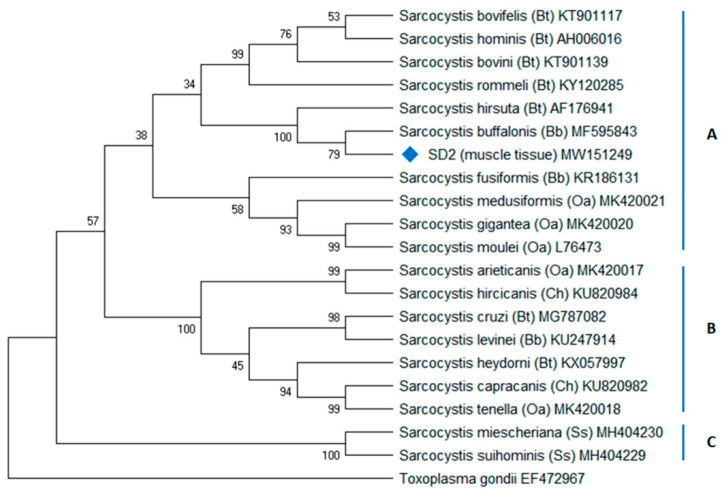
Phylogenetic positioning of the *Sarcocystis*-like organism found in muscle of striped dolphin (*Stenella coeruleoalba*) SD2 in Liguria, Italy. The evolutionary history was inferred using the maximum parsimony (MP) method. Tree n.1 out of three most parsimonious trees (length = 1532) is shown. The consistency index is (0.750000), the retention index is (0.820830), and the composite index is 0.689026 (0.615623) for all sites and parsimony-informative sites (in parentheses). The percentage of replicate trees in which the associated taxa clustered together in the bootstrap test (1000 replicates) is shown next to the branches [29].The MP tree was obtained using the subtree–pruning–regrafting (SPR) algorithm [30] with search level 1 in which the initial trees were obtained by the random addition of sequences (10 replicates). This analysis involved 21 nucleotide sequences from *Sarcocystis* species infecting domestic hosts that are raised in Europe (Bt, *Bos taurus*; Bb, *Bubalus bubalis*, Ch, *Capra hircus*; Oa, *Ovis aries*; Ss, *Sus scrofa*). In cluster B and C, a high or moderate bootstrap (BP) value at each node supported each group containing closely related *Sarcocystis* species with canids as the definitive host, respectively, whereas, in cluster A, some low BP values indicated that the phylogenetic position of *Sarcocystis* species with felids as definitive hosts is not conclusive (BP = 34–38%). These results are probably due to the fact that the 18S rRNA locus is not the most appropriate to infer phylogenetic relationships; moreover, the short length of the SD2 sequence obtained is a limitation identified here. Nonetheless, it should be noted that neighbor-joining and maximum-likelihood methods also resulted in phylogenetic trees in which *S. hirsuta*, *S. buffalonis*, and SD2 *Sarcocystis* sp. grouped together (data not shown). There were a total of 1974 positions in the final dataset. Evolutionary analyses were conducted in MEGA X [26].

Phylogenetic analysis of the 18S rRNA *Sarcocystis* sp. sequence from SD2 indicated a *Sarcocystis buffalonis*-like organism (MW151249) when the sequence was compared to genetically similar species and the *Sarcocystis* spp. infecting major livestock species in Mediterranean Europe (Figure 4). The *S. hirsuta*-like sequence (MW151248) was not included in the tree because of its short length (122 bp). PCR n.11 screening for *N. caninum* DNA resulted in none detected in both cases. All SD2 target organs tested for the presence of *T. gondii* DNA were strongly positive (100% homology with other *T. gondii* sequences deposited such as MH793505). Furthermore, all three DNA samples from SD2 were genotyped by PCR-RFLP method as ToxoDB genotype #3 showing type II alleles for all the markers except *Apico* (type I allele) (Table 2).

## 4. Discussion

NS meningoencephalitis in dolphins is usually related to *B. ceti* infections, to viruses such as DMV and HV, and to protozoa, especially *T. gondii* [7,31]. In this study, involving two striped dolphins (*Stenella coeruleoalba*) stranded along the Ligurian coast of Italy, samples from SD2 demonstrated the presence of *T. gondii*, DMV, and *B. ceti*. This coinfection, alongside with the toxicological stress detected (CAN ˃ 0.47, [18]), has been considered responsible for the cerebral impairment and the consequential animal’s stranding [10]. In contrast, samples from SD1 lacked direct detection of commonly recognized neurotropic agents to explain the observed neuroinflammatory pattern present. SD1 demonstrated serological evidence of DMV infection, evident by a very low titer of antibodies, and suggestive of contact with the virus, rather than the disease, i.e., subclinical infection [32]. Therefore, a closer examination of the tissues for other potential neurotropic agents was undertaken for SD1 and SD2 for comparison.

From the histopathological examination, the morphological appearance of the unusual cysts observed in both animals was highly suggestive of a *Sarcocystis*-like coccidium. Although neither villous protrusions nor internal septations were observed, the protozoan tissue cysts were large (up to 116 μm in diameter), presented with a discernible thin outer wall, and the enclosed bradyzoites stained more basophilic than *T. gondii* ones.

As it is routinely done in suspected cases of toxoplasmosis, an immunohistochemical characterization of the unknown organism was attempted. The obtained results were inconclusive and consistent with the reviewed literature [20,21,22]. The reactivity of the two polyclonal *Sarcocystis* spp. antisera was mild and failed to label the tissue cyst wall. The absent reactivity of the cyst wall may be related to its maturity, as previously proposed [22]. This similar reactivity of both the anti-*S. neurona* and anti-*S. falcatula* antisera to the cysts is in agreement with what has been previously reported [20,21]. A possible broader spectrum of antigenic cross-reactivity between closely related *Sarcocystis* species and other Apicomplexa has been suggested [20,33].

It should be also noted that, during previous standard investigations, large *Sarcocystis*-like tissue cysts in the CNS of both animals stained positively to the anti-*T. gondii* PoAb, even in the absence of a molecular and serological confirmation of *T. gondii* in SD1. This finding suggests that common epitopes may be shared between these two protozoa and that the commercial anti-*T. gondii* PoAb is not specific in discriminating between these two protozoa, as previously documented by other authors [34], in cases of closely related cyst-forming apicomplexan parasites. The anti-*S. neurona* 2G5 monoclonal antibody did not stain the bradyzoites or the cyst wall of the protozoan tissue cyst in SD1 brain. Although this monoclonal antiserum is directed against a more conserved epitope among *S. neurona* strains and it is suitable to stain FFPE tissues [21], low reactivity was also observed by other authors [35] in the IHC investigations performed on the brains from California Sea otters (*Enhydra lutris nereis*) with PCR-confirmed *S. neurona* infection.

Therefore, the diagnostic value of the IHC staining is questionable and, in this study, could be limited in that both the monoclonal and polyclonal antibodies were raised against merozoite epitopes and may not be suitable to label the cyst wall or the enclosed bradyzoites [35].

As the IHC evaluations were insufficient for parasite identification, in order to have a final confirmation of the parasites detected, 11 different PCR protocols were employed in an attempt to molecularly characterize the tissue-cyst forming protozoa. *Sarcocystis* sp. infections, previously unreported in muscle tissue of Mediterranean cetaceans, were confirmed by PCR means in the myocardium and in the skeletal muscle.

PCR n.1 excluded parasite identification as *S. neurona* or *S. falcatula* in either SD1 or SD2. Moreover, the sequencing of the 18S rDNA amplicon to discern the presence of mixed protozoal infections [35] was nonspecific in SD1, resulting in exclusion of other known tissue cyst-forming coccidia, such as *T. gondii*, *N. caninum*, and *Hammondia hammondi*. *Neospora caninum* infection was also excluded by specific PCR n.11, and the thickness of the observed tissue cyst walls did not correspond to what is expected in such an organism.

The results obtained from SD2 tissues should be carefully interpreted. A majority of the DNA sequencing showed homology to *T. gondii* sequences deposited in GenBank^®^. SD2 was already proven coinfected with *T. gondii* by means of PCR [14] and IHC [6] and the massive infection by *T. gondii* could have masked the detection of other pathogens, including *Sarcocystis* sp. Perhaps, in SD2, DNA extraction from purified tissue cyst would be more effective as compared to genomic DNA extracted without isolating tissue cyst from the surrounding tissue [36].

PCR protocol n.6 succeeded in amplifying *Sarcocystis* sp. DNA sequences from target organs. A short sequence (122 bp), showing high BLAST homology with *S. hirsuta*, was obtained from SD1 myocardium, whereas a longer (188 bp), high-quality sequence was obtained from SD2 skeletal muscle showing 97.8% homology with *S. buffalonis* isolates.

To date, *S. hirsuta* and *S. buffalonis* have been reported only in cattle and water buffaloes [33] and seem to be strictly intermediate host-specific, like other livestock *Sarcocystis* species, with no reported infection in non-ruminant intermediate hosts. Furthermore, in the present study, parasite identification was achieved targeting the 18S rRNA gene but other authors [37,38] recommend the analysis of *cox1* gene to molecularly discriminate between *Sarcocystis* species-infecting hosts from the Bovidae family.

Although lacking additional natural cases or experimental infections, the possibility for marine wildlife to share *Sarcocystis* species with domestic animals cannot be ruled out completely, especially when intermediate hosts are phylogenetically related. Specifically, the order Cetacea is a sister-group to the family Hippopotamidae and to the Ruminantia taxa [39] which includes also the Bovidae family.

However, considering the results of the BLAST analysis in relationship to sequence quality and the host specificity of this protozoal family, it is most likely that a previously-undescribed *Sarcocystis*-like protozoa, within the family Sarcocystidae and phylogenetically related to species cycling in livestock, is infecting Mediterranean marine mammals. The lack of reports and of prevalence data on muscular sarcocystosis in the Mediterranean makes it difficult to determine the origin of this pathogen.

Since the highest degree of similarity was observed with bovine and bubaline *Sarcocystis* species, a land-to-sea transfer can be hypothesized for this protozoon even though, in the absence of an exhaustive characterization, a marine cycle cannot be discarded. Being that most *Sarcocystis* species are highly host-specific, the hypothesis of a two-host heteroxenous marine cycle, like the one proposed for *Sarcocystis balaenopteralis* [40], seems unlikely as dolphins are apex predators rather than prey in the Mediterranean food web.

To date, *S. neurona* is commonly reported in cases of muscular and neural sarcocystosis diagnosed in marine mammals in North America [1], but its presence has not yet been reported in Europe. However, a *S. neurona* infection in Mediterranean dolphins is highly improbable due to the lack of the definitive hosts (*D. virginiana* and *D. albiventris*) which are geographically confined to the New World.

In the Mediterranean basin, protozoal meningoencephalitis has been limited to reports associated with *T. gondii* subacute to chronic infections [6,7]. A *T. gondii* infection was also confirmed and fully characterized by means of TEM, IHC, and PCR in the present study. Multilocus RFLP PCR genotyping of the *T. gondii* strain infecting SD2 retrieved a type II PRU variant genotype (ToxoDB#3), which is common in felids, livestock, and wildlife around Europe [41,42]. To date, type II genotypes account for the totality of toxoplasmosis reports in Mediterranean cetaceans [43,44,45] (reviewed in Table 3). Although a marine cycle for this parasite cannot be precluded, the results from SD1 and SD2 support the role of protozoa as a land-base “pollutant” that have expanded the range of their intermediate hosts to the marine environment.

Previously, *Sarcocystis* spp. have not been observed in histopathological brain sections of stranded cetaceans. The only documented *Sarcocystis* sp. infection in a wild Mediterranean marine mammal is a hepatic sarcocystosis due to a *S. canis*-like protozoan infecting a striped dolphin stranded along the Spanish coast [8]. *Sarcocystis* spp. have already been reported in other marine mammals [50,51]. In aquatic species, *S. canis*-like infection causes a fatal and acute hepatitis with microscopic lesions confined to the liver [8,24,50]. Mature and immature schizonts are the protozoal stages observed during histopathological and TEM investigations, whereas tissue cysts have not been observed [8,50].

However, sarcocysts in muscle tissue are chronic lesions that have been incidentally observed in mysticetes and odontocetes from other geographical areas without associated pathology [52,53,54,55,56]. In previous reports, protozoal tissue cysts were attributed to *Sarcocystis* spp. only on the basis of light microscopy and TEM findings. In our study, muscular sarcocystosis was evidenced by means of PCR in each stranded dolphin. To the authors’ best knowledge, no other *Sarcocystis* species have been previously observed or isolated in Mediterranean cetaceans. Moreover, the prevalence of muscular sarcocystosis in Mediterranean marine mammals is unknown as such infections are likely overlooked during routine investigations. Therefore, it is not possible to state whether or not tissue cysts are a common finding in muscle tissues and which species of *Sarcocystis* are prevalent in Mediterranean marine mammals.

The most likely hypothesis would be to consider the same protozoa in the muscle tissues as the etiologic agents of the cysts observed in the CNS. Nevertheless, we cannot discard the possibility of two different species infecting the same host. The morphology of the observed neural cysts is highly suggestive of a *Sarcocystis*-like coccidium. It was not feasible to perform a morphological comparison with the muscle tissue cysts because SD1 FFPE skeletal muscle was not available and only *T. gondii* muscular cysts were observed in SD2 skeletal muscle sections (*data not shown*), while no cysts were identified in the heart sections of either SD1 or SD2.

As the biomolecular investigations failed in amplifying specific *Sarcocystis* sequences in the CNS, further investigations are needed to confirm our putative diagnosis.

## 5. Conclusions

The present study represents the first description of a *Sarcocystis*-like infection in muscle tissue of dolphins from the Mediterranean basin along with the first report of *Sarcocystis*-like tissue cysts in the brain of stranded cetaceans.

The *T. gondii* strain detected belongs to a common genotype circulating in Europe, while the unknown organisms were genetically similar to *Sarcocystis* species infecting the Bovidae family. Such results might suggest a land-to-sea cycling of these Apicomplexan parasites and the need for further investigations.

Because of the novelty of these findings, special attention should be reserved for the differential diagnosis of protozoal infections when performing sanitary surveillance on stranded Mediterranean cetaceans, including collection and preservation of tissues to enable a panel of characterization studies.

## Figures and Tables

**Table 1:** Stranding data, body condition, most significant findings (gross and microscopic), pathogens detected, and the hypothesis of causa mortis in the two animals under study.

ID	YS	DC	NuS	Age/ Sex	Main Lesions (Gross and Microscopic)	Detected Pathogens	Cause of Death	Reference
SD1	2011	3	Poor	Adult M	Severe granulomatous pneumonia; fibrinous peritonitis; splenomegaly associated to chronic granulomatous splenitis; cholangiohepatitis; generalized lymphadenitis associated to lymphoid depletion; severe NS meningoencephalitis	*Photobacterium damselae* subsp. *damselae* (isolated from blowhole and lungs); *Campula palliata* (pancreas and liver); *Monorygma grimaldi* (musculature and peritoneum); *Skrjabinalius guevarai* (lung); anti-DMV antibodies (1:16) in blood serum (VN)	Infectious disease (parasitic and unknown agent)	[11]
SD2	2017	2	Moderate	Juvenile M	Skin ulcers; ulcerative glossitis; subcutaneous parasitic cysts; bronchointerstitial pneumonia; multifocal necrotizing hepatitis; cholangiohepatitis; splenomegaly and generalized lymphadenomegaly associated to multicentric lymphoid necrosis; interstitial nephritis; lymphadenitis; severe NS meningoencephalitis	*Phyllobotrium* spp (blubber); *Monorygma grimaldii* (musculature); *Brucella ceti* (isolation from CNS, lung, and spleen; PCR from brain, liver, lung spleen, and lymph nodes); DMV (PCR, IHC from CNS, spleen, kidney, tonsils, and lymph nodes, bladder and muscle); *Toxoplasma gondii* (PCR, IHC from CNS, lymph nodes, spleen, liver, and muscle); anti-DMV antibodies (1:8) in blood serum (VN); anti-*T. gondii* antibodies (>1:640) in blood serum, (1:160) in CSF, (1:80) in aqueous humor (IFAT). Severe immunosuppression (CAN = 0.688) [18]	Infectious disease (viral, bacterial, and parasitic)	[10]

YS = year of stranding; DC = decomposition code (2, fresh; 3, moderate autolysis); NuS = nutritional status; M = male; NS = nonsuppurative; DMV = *Dolphin morbillivirus*; VN = virus neutralization; CNS = central nervous system; PCR = polymerase chain reaction; IHC = immunohistochemistry; CFS = cerebrospinal fluid; IFAT = indirect fluorescent antibody technique; CAN = canonical variable.

**Table 2:** Results of genotyping analysis carried out on *Toxoplasma gondii* strain identified in dolphin SD2.

Isolate/ Sample	SAG1	3′-SAG2	5′-SAG2	Alt. SAG2	SAG3	BTUB	GRA6	c22-8	C29-2	L358	PK1	Apico	ToxoDB PCR-RFLP Genotype #
**RH** **(ref. type I)**	I	I/III	I/II	I	I	I	I	I	I	I	I	I	#1
**Me-49** **(ref. type II)**	II/III	II	I/II	II	II	II	II	II	II	II	II	II	#1
**NED** **(ref.** **type III)**	II/III	I/III	III	III	III	III	III	III	III	III	III	III	#2
**SD2** **(muscle)**	II/III	II	I/II	II	II	II	II	II	II	II	II	I	#3
**SD2 (CNS)**	II/III	II	I/II	II	II	II	II	II	II	II	II	I	#3
**SD2 (Heart)**	II/III	II	I/II	II	II	II	II	II	II	II	II	I	#3

**Table 3:** Summary of the available literature reporting genotyping data on *Toxoplasma gondii* strains infecting dolphins.

Host	Location	N°. of Individuals	Condition	Genotype (n)	Method (Markers)	Isolate ID	Reference
Bottlenose dolphin (*Tursiops truncatus*)	South Carolina (USA)	3	Stranded	#1 (2/3); Unique (1/3)	PCR-RFLP (SAG1, SAG2, SAG3, BTUB, GRA6, c22-8, c29-2, L358, PK1, and Apico)	TgDoUs1-3	[46]
	Canada (born in Russia)	1	Captivity	#3 (1/1)	PCR-RFLP (B1, SAG1, SAG2, SAG3, BTUB, GRA6, c22-8, c29-2, L358, PK1, and Apico) +PCR-Seq (B1, SAG1)	TgDoCA1	[47]
Hector’s dolphins *(Cephalorhynchus hectori)*	New Zealand	8	By caught/ stranded	#3 (7/8); Type II variant (Type I + II at L358 and Type I at Apico)	PCR-RFLP (SAG1, SAG2 (5‘ + 3‘), SAG3, GRA6, L358, PK1, and Apico)	No isolation	[48]
Striped dolphin (*Stenella coeruleoalba*)	Costa Rica	1	Stranded	#1 (1/1)	PCR-RFLP (SAG1, SAG2, SAG3, BTUB, GRA6, c22-8, c29-2, L358, PK1, and Apico)	TgSdCo1	[49]
	Italy	3	Stranded	Type II (2/3); Unique (1/3)	PCR-seq (B1, gra6 a nd uprt1)	TSL2, TSL3, and TSL6	[43]
	Italy	1	Stranded	#1 (1/1)	RFLP-PCR (SAG1, SAG2 (5‘ + 3‘), alt SAG2, SAG3, BTUB, GRA6)	No isolation	[45]

## Data Availability

The data presented in this study are available within the article and the Supplementary Materials. The sequences generated in the present study were submitted to the GenBank database with the following accession numbers: MW151248-MW151249.

## Supplementary Materials

The following are available online at https://www.mdpi.com/article/10.3390/ani11051201/s1, Table S1: Selected PCR protocols used in the present study to detect DNA from tissue-cyst forming coccidian, Table S2: Results of the PCR protocols screening for tissue cyst-forming coccidian DNA in target organs.
