# Supplementary material for: Evidence for Unknown Sarcocystis-Like Infection in Stranded Striped Dolphins (Stenella coeruleoalba) from the Ligurian Sea, Italy
Source: Animals (Basel). 2021 Apr 22;11(5):1201. doi: 10.3390/ani11051201 (PMC8143450; doi:10.3390/ani11051201)
Supplement: Supplementary file 1 [file animals-11-01201-s001.zip › animals-1171546-supplementary.pdf]

animals MDPI

Supplementary material Table S1. Selected PCR protocols used in the present study to detect DNA from tissue-cyst forming coccidia.

Protocol	Organism	Target region (size, bp)	Primers name	Reference
**1**	Tissue-cyst forming coccidia	*18S rDNA* (344/396)	18S FwdExt/18S RevExt/18S RevInt	[29]
	S. neurona and *S. falcatula*	*ITS-1* (500)	ITS1$_{500}$ FwdExt/ITS1$_{500}$ RevExt/ITS1$_{500}$ FwdInt/ITS1$_{500}$ ReveInt	
**2**	*Sarcocystis* spp.	*ITS-1* (1025-1030)	ITS-5/ITS-2	[30]
**3**	*Sarcocystis* spp.	*ITS-1* (700)	ITS-5/ITS-SmRi	[30]
**4**	*Sarcocystis* spp.	*ITS-1* (426)	ITS-SmFi/ITS-SmRi	[30]
**5**	*Sarcocystis* spp.	*Sarcocystis* spp. specific product (334)	JNB25/JD396	[31]
**6**	*Sarcocystis* spp.	18S rRNA (164/186)	SARf/SARf	[32]
**7**	*Sarcocystis* spp.	*cox1* (1038)	SF1/SR9	[33]
**8**	*Sarcocystis* spp.	*cox1* (1012)	SF1/SR5	[34]
**9**	*Sarcocystis* spp.	*18S rRNA* (950)	2L/2H/3H	[35]
**10**	*T. gondii*	*ITS-1* (227)	Tg-NN1/Tg-NN2/Tg-NP1/Tg-NP2	[36]
**11**	*N. caninum*	*Nc5* (350)	Np21+/Np6+	[37]

Supplementary material Table S2. Results of the PCR protocols screening for tissue-cyst forming coccidia DNA in target organs.

**Case**		**SD1**			**SD2**		
**Target tissue**		Heart (FFPE)	Muscle	Brain (FFPE)	Heart	Muscle	Brain
**PCR 1**	**18S primers**	+	+	+	+	+	+
	**$ITS1_{500}$ primers**	—	—	—	—	—	—
**Seq.**		NS	NS	NS	*T. gondii*	*T. gondii*	*T. gondii*
**PCR 2**		—	—	—	—	—	—
**PCR 3**		—	—	—	—	—	—
**PCR 4**		—	—	—	—	—	—
**PCR 5**		+	+	+	+	+	+
**Seq.**		NS	NS	NS	NS	NS	NS
**PCR 6**		+	—	—	—	+	—
**Seq.**		*S. hirsuta* (99.4%)	NA	NA	NA	*S.buffalonis* (97.8%)	NA
**PCR 7**		—	—	—	+	—	+
**Seq**		NA	NA	NA	NS	NA	NS
**PCR 8**		—	—	—	+	—	+
**Seq.**		NA	NA	NA	*T. gondii*	NA	*T. gondii*
**PCR 9**		—	—	+	+	+	+
**Seq.**		NA	NA	NS	*T. gondii*	*T. gondii*	*T. gondii*
**PCR 10**		-	-	-	+	+	+
**Seq.**		NA	NA	NA	*T. gondii*	*T. gondii*	*T. gondii*
**PCR 11**		—	—	—	—	—	—

Legend: Seq: sequencing; NS: not specific; NP: not performed; NA : not applicable
